# Targeted therapy for Wilms tumor: a bibliometric study of worldwide scientific activity and collaborative networks

**DOI:** 10.1007/s12672-025-03255-y

**Published:** 2025-07-25

**Authors:** Boshen Shu, Shufeng Zhang, Jian Gao, Lin Wang, Xiaohui Wang

**Affiliations:** Department of Pediatric Surgery, Henan Provincial People’s Hospital, Zhengzhou, 450003 Henan Province China

**Keywords:** Wilms tumor, Nephroblastoma, Bibliometrics, Global collaboration, Visualized analysis, VOSviewer

## Abstract

**Background and purpose:**

Wilms tumor (WT), or nephroblastoma, is the most common abdominal solid tumor in children with a recurrence rate as high as 15%. Targeted therapy is an effective treatment choice for patients with WT. This study aimed to evaluate the global research activity and collaborative networks of WT targeted therapy through the bibliometric analysis.

**Methods:**

The Web of Science Core Collection (WoSCC) database was used to search related studies on targeted therapy for WT published between 1945 and 2024. Subsequently, the VOSviewer, Graphpad, and Bibliometrix package in the R Studio were applied to conduct bibliometric and visualized analysis.

**Results:**

A total of 1,604 publications were included in our analysis. The USA (*n* = 572) took the dominant position in the number of publications and Harvard University contributed most papers (*n* = 122). Sugiyama H and Oka Y demonstrated superior performance in this domain, with Sugiyama H leading in the number of publications (*n* = 59) and Oka Y leading in terms of co-citations (*n* = 382). The most productive journal was the *Oncogene* (*n* = 44). “Expression” and “Wilms Tumor” were the most frequent keywords, while “lncRNA”, “Biomarkers” and “microRNA” were recent hotspots.

**Conclusions:**

Research on targeted therapy for WT has developed rapidly with increasing interests, which emphasizes its growing importance in the scientific community. Nonetheless, the primary research has been primarily concentrated in a limited number of developed regions, and global collaboration remains inadequate. International collaborations and translational research should be reinforced to facilitate further advancements in this field.

## Introduction

Wilms tumor (WT), also known as nephroblastoma, is the most common renal tumor in pediatric population, accounting for more than 90% of renal tumors in children [1]. WT has been observed in 1 in 10,000 children in North America, typically manifesting before the age of five and exhibiting equal prevalence among both genders [2]. Most children diagnosed with WT present to medical care following their parents’ detection of palpable abdominal lumps. In some cases, additional symptoms may be present, including hematuria, fever, urinary tract infection, varicocele and others [3]. WT demonstrates a predominantly ​​sporadic etiology​​, with ​​familial cases accounting for only 1–2%​​ of all occurrences [4]. Familial cases are relevant to a higher occurrence of bilateral tumors along with a lower diagnosing age [5]. Significant progress has been made in the management of WT in recent years, the 5-year survival rate of children with WT is approximately 90% [6]. Nonetheless, previous research has demonstrated the recurrence rate of WT is around 15% and the long-term survival rate of relapsed WT is about 40–70% [7, 8]. Significant challenges persist in advancing multimodal therapeutic approaches for WT that combine surgical intervention, chemotherapy and radiotherapy. Targeted therapy has improved the accuracy of anti-tumor activity and reduced side effects, which has played an indispensable role in WT treatment. Given the proliferation of studies in this field and broad spectrum of topics, it is essential to conduct a quantitative and qualitative analysis of global WT targeted therapy research.

Bibliometric analysis is formally defined as the quantitative and qualitative evaluation of published literature through statistical examination of factors such as citation, distribution, impact, collaboration and more. Coined by Alan Pritchard in 1969, its core objective remains the systematic mapping of disciplinary landscapes to identify research frontiers, emerging trends and scholarly impact [9]. Recent trends have observed an increased utilization of bibliometrics benchmarks by stakeholders and grant authorities in the evaluation of researchers’ performance for the purpose of determining academic appointment candidates or funding eligibility [10, 11]. It has been broadly used in many research fields to assess the influence of published literature or identify research frontiers due to these advantages [9, 12, 13]. To date, no bibliometric study has been conducted to analyze the research activity pertaining to WT targeted therapy, therefore, we aimed to comprehensively evaluate the global research activity and future trends in this field to provide valuable insights into the development of WT targeted therapy research.

## Methods

### Data source and search strategy

**Fig. 1:**
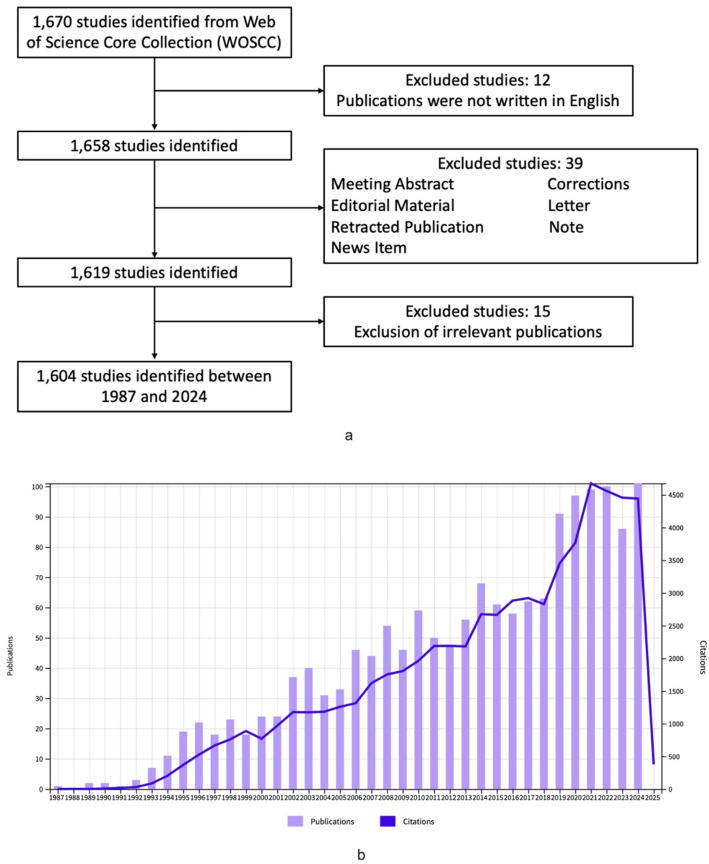
**a** Screening process of relevant publications in the field of WT targeted therapy. **b** Publication and citation trends on WT targeted therapy between 1987 and 2024

Despite the existence of numerous alternative databases capable of facilitating literature searches and bibliometric analysis, such as Scopus and Google Scholar, Web of Science Core Collection (WoSCC) database was used as the optimal database for this investigation due to its widespread reputation as the most suitable platform for bibliometrics [14]. The relevant publications were obtained from the WoSCC database by using the following strategies: TS = (Targeted OR Targeting OR Targeted therapy OR Targeted Treatment OR Targeting Treatment) AND (Nephroblastoma OR Wilms tumor OR Wilms’ tumor). In this study, only articles and reviews written in English were included. The retrieved results were saved as “Full Record and Cited References” and “Plain Text” formats, which were analyzed subsequently. To avoid bias, two independent authors searched the literature, with a third author reviewing the results. All searches were conducted on the same day (February 06, 2025) to ensure reliability. The retrieval strategy process was depicted in Fig. 1a.

### Data analysis

The following factors were recorded and subsequently analyzed: literature title, corresponding journal, author, published year, total citations, institution, country, keyword, impact factor (IF) and h-index. The h-index is considered as an objective parameter for assessing the scientific impact of research from levels of authors, institutions or countries. Leveraging quantitative analytical methodologies, this index facilitates the estimation of the impact of a particular research contribution [15]. VOSviewer, developed by the Leiden University, is applied for visualizing and analyzing collaboration networks among publications in different research fields [14, 16]. The VOSviewer (version: 1.6.20) and Bibliometrix package (version: 4.1.1) based on R Studio were applied to explore collaboration network for authors, institutions and countries in our study. While for creating network visualization maps to identify the current research hotspots and future potential trends, keyword co-occurrence analysis and the overlay visualization were utilized. In the bibliometric network visualizations, entities (authors, countries, institutions, keywords) are represented as nodes, while collaborative relationships between these entities are quantified through connecting links [17].

## Results

### Global research productivity

After screening, a total of 1604 publications relevant to WT targeted therapy were included, of which 1389 research articles and 215 reviews. These selected publications were from 74 countries, 1909 institutions, 589 journals and 8,954 authors. The number of publications and citations per year increased from 1987 to 2024, from one and zero, respectively, to 101 and 4443 (Fig. 1b). A total of 64,800 citations were recorded and 58,146 of which did not include self-citations. The average citation was 40.4 and the total h-index was 119.

### Analysis of countries and institutions

**Fig. 2:**
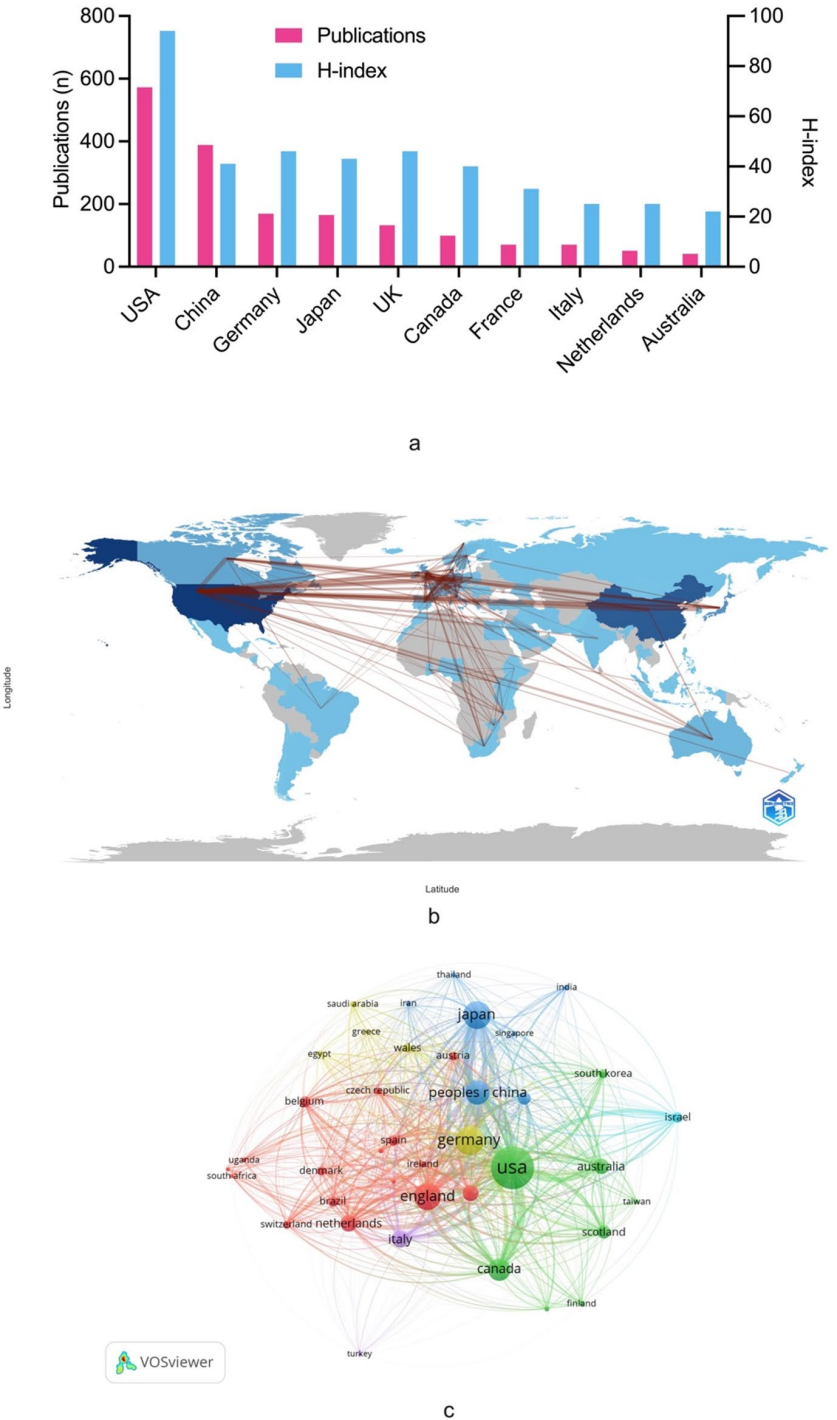
**a** The top ten productive countries and their related h-index regarding WT targeted therapy. **b** International collaboration of WT targeted therapy research. **c** Bibliographic coupling analysis of global collaboration in WT targeted therapy research

In total, 74 countries contributed to the research of targeted therapy for WT, of which the top ten leading countries regarding publication number were presented in Fig. 2a. The USA owned the highest number of publications (*n* = 572, 35.7%, h-index = 94), followed by China (*n* = 388, 24.2%, h-index = 41) and Germany (*n* = 169, 10.5%, h-index = 46). Research output concentrated predominantly in North America and Europe, accounting for the majority of the top ten most productive countries. The global collaboration network maps provided clear worldwide collaboration connections at country and continent levels, with the most frequent connections showing the strongest relationships among North America, Europe and East Asia (Fig. 2b). While for the country level, the USA, the UK, China, Germany and Japan showed frequent associations (Fig. 2c).

**Fig. 3:**
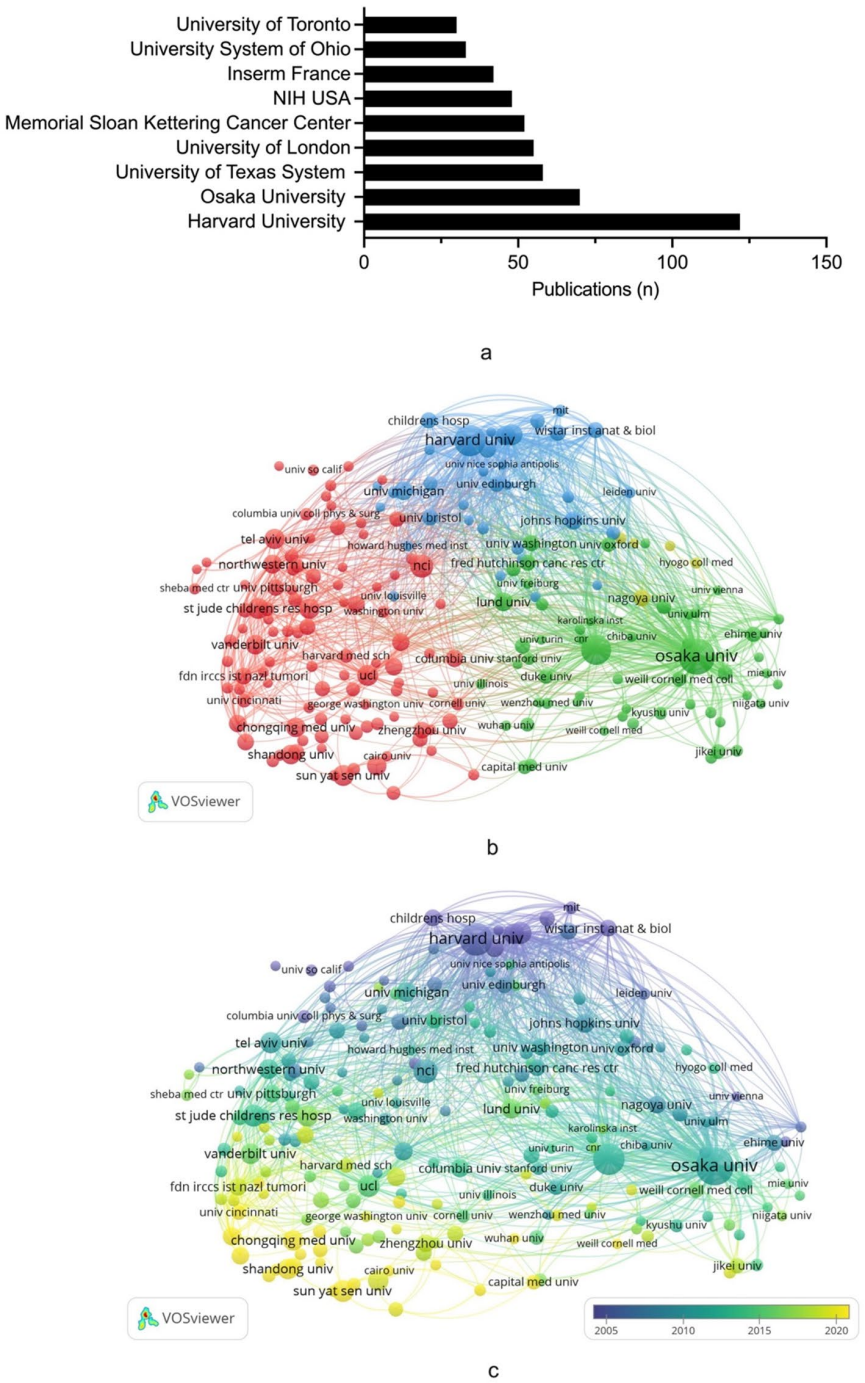
**a** The top ten contributing institutions on WT targeted therapy. **b** Collaboration network among institutions worldwide. **c** Visualization of the institution collaboration appearing time according to overlay visualization. The institutions highlighted in yellow appeared later than those highlighted in green

Among the 1909 institutions, Harvard University contributed most papers (*n* = 122, 7.6%), followed by Osaka University (*n* = 70, 4.4%) and University of Texas System (*n* = 58, 3.6%) (Fig. 3a). Osaka University dominated the institutional collaboration network with the strongest total link strength (*n* = 81,088), significantly exceeding Harvard University (*n* = 54,465) and Memorial Sloan-Kettering Cancer Center (*n* = 37,462) (Fig. 3b). Remarkably, several institutions such as Chongqing Medical University and Cairo University from developing countries also showed notable collaboration relationships in recent years (Fig. 3c).

### Analysis of authors, journals and impactful references

**Fig. 4:**
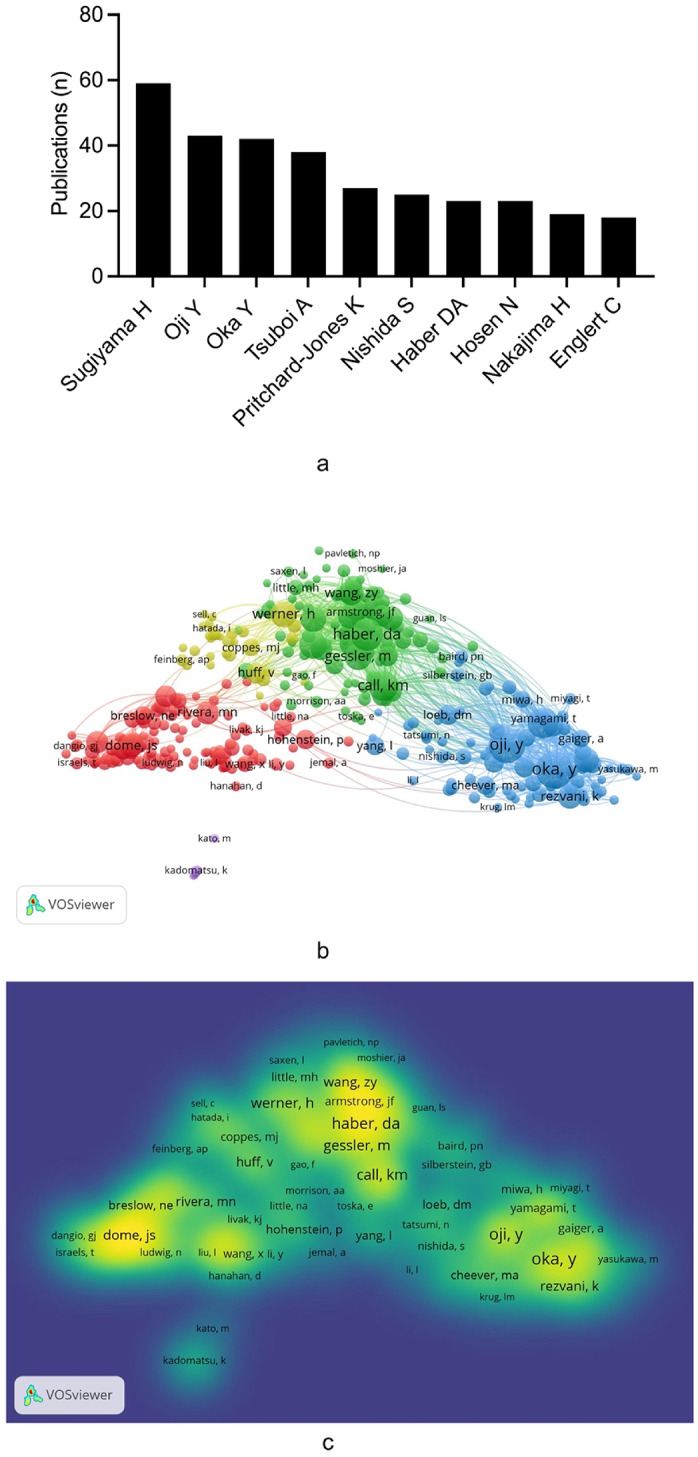
**a** The top ten authors with the highest number of publications in the research of WT targeted therapy. **b** Analysis of co-cited author collaboration network in WT targeted therapy research. **c** Mapping on density visualization of co-cited author collaboration in WT targeted therapy research. Different colors represent different frequencies of collaboration. Blue indicates low frequency, green indicates medium frequency, and yellow indicates high frequency. Interactive collaboration in a yellow circle is more closely related than those in other colored circles

A thorough examination of the authors and their collaboration networks can yield significant insights into the cooperation between prominent researchers in the field of WT targeted therapy. This analysis will also facilitate a more comprehensive understanding of the current state of research and frontiers in this domain. The present study involved a total of 8,954 authors, Sugiyama H (*n* = 59, 3.7%) emerged as the most prolific scholar, followed by Oji Y (*n* = 43, 2.7%) and Oka Y (*n* = 42, 2.6%) (Fig. 4a). While for the network graph of co-cited author analysis, Oka Y led in terms of co-citations (*n* = 382), followed by Oji Y (*n* = 364) and Haber DA (*n* = 287) (Fig. 4b, c). Notably, Oka Y, Oji Y and Haber DA demonstrated ​​dual prominence​​, simultaneously appearing in both the ​​top ten most published​​ and ​​most co-cited author lists​​.

Studies relevant to WT targeted therapy were published in 589 journals, and the top ten journals and co-cited journals were displayed in Tables 1 and 2, respectively. *Oncogene* contributed the most of publications (*n* = 44), followed by *Journal of Biological Chemistry* (*n* = 36) and *Pediatric Blood & Cance*r (*n* = 26). For the active journals in the co-cited journal analysis, *Blood* led the way regarding the number of co-citations (*n* = 2,850), followed by *PNAS* (*n* = 2588) and *Cancer Research* (*n* = 2497). Significantly, *Oncogene*, *Journal of Biological Chemistry*, *PNAS* and *Cancer Research* ranked among the top ten journals for both productivity and co-citation frequency.

**Table 1:** The top ten productive journals on the research of WT targeted therapy

Rank	Journals	Publications (*n*)	Citations (*n*)	IF	JCR-c	Country
1	Oncogene	44	2316	6.9	Q1	UK
2	Journal of Biological Chemistry	36	1923	4	Q2	USA
3	Pediatric Blood & Cancer	26	464	2.4	Q2	USA
4	Anticancer Research	25	400	1.6	Q4	Greece
5	Cancer Research	21	1672	12.5	Q1	USA
6	Leukemia	21	1291	12.8	Q1	UK
7	Frontiers in Oncology	19	235	3.5	Q2	Switzerland
8	PNAS	19	2926	9.4	Q1	USA
9	PLoS One	18	434	2.9	Q1	USA
10	Cancers	17	103	4.5	Q1	Switzerland

**Table 2:** The top ten co-cited journals on the research of WT targeted therapy

Rank	Co-cited journals	Co-citations (*n*)	IF	JCR-c	Country
1	Blood	2850	21.1	Q1	USA
2	PNAS	2588	9.4	Q1	USA
3	Cancer Research	2497	12.5	Q1	USA
4	Nature	2261	50.5	Q1	UK
5	Cell	2171	45.6	Q1	USA
6	Journal of Biological Chemistry	2047	4	Q2	USA
7	Oncogene	1674	6.9	Q1	UK
8	Science	1498	44.8	Q1	USA
9	Journal of Clinical Oncology	1390	42.1	Q1	USA
10	Clinical Cancer Research	1314	10.4	Q1	USA

The co-cited references for WT targeted therapy reached a total of 52,555, with 142 of them being cited more than 30 times. Table 3 showed the top ten co-cited references, among which 3 publications have been cited more than 150 times. The reference with the highest number of citations was “Isolation and characterization of a zinc finger polypeptide gene at the human chromosome 11 Wilms’ tumor locus” published in 1990 by Call et al. in *Cell* [18].

**Table 3:** The top ten co-cited references on the research of WT targeted therapy

Rank	Title	Journal	Author	Year	Citations (*n*)	IF	JCR-c
1	Isolation and characterization of a zinc finger polypeptide gene at the human chromosome 11 Wilms’ tumor locus	Cell	Call et al.	1990	249	45.6	Q1
2	WT-1 is required for early kidney development	Cell	Kreidberg et al.	1993	187	45.6	Q1
3	Homozygous deletion in Wilms tumours of a zinc-finger gene identified by chromosome jumping	Nature	Gessler et al.	1990	163	50.5	Q1
4	The candidate Wilms’ tumour gene is involved in genitourinary development	Nature	Pritchard-Jones et al.	1990	117	50.5	Q1
5	Expression of the interleukin-6 (IL-6), IL-6 receptor, and gp130 genes in acute leukemia	Blood	Inoue et al.	1994	115	21.1	Q1
6	Alternative splicing and genomic structure of the Wilms tumor gene WT1	PNAS	Haber et al.	1991	115	9.4	Q1
7	Induction of WT1 (Wilms’ tumor gene)-specific cytotoxic T lymphocytes by WT1 peptide vaccine and the resultant cancer regression	PNAS	Oka et al.	2004	110	9.4	Q1
8	The prioritization of cancer antigens: a national cancer institute pilot project for the acceleration of translational research	Clinical Cancer Research	Cheever et al.	2009	95	10.4	Q1
9	Binding of the Wilms’ Tumor Locus Zinc Finger Protein to the EGR-1 Consensus Sequence	Science	Rauscher et al.	1990	89	44.8	Q1
10	Repression of the Insulin-Like Growth Factor II Gene by the Wilms Tumor Suppressor WT1	Science	Drummond et al.	1992	88	44.8	Q1

### Analysis of keywords and future trends

**Fig. 5:**
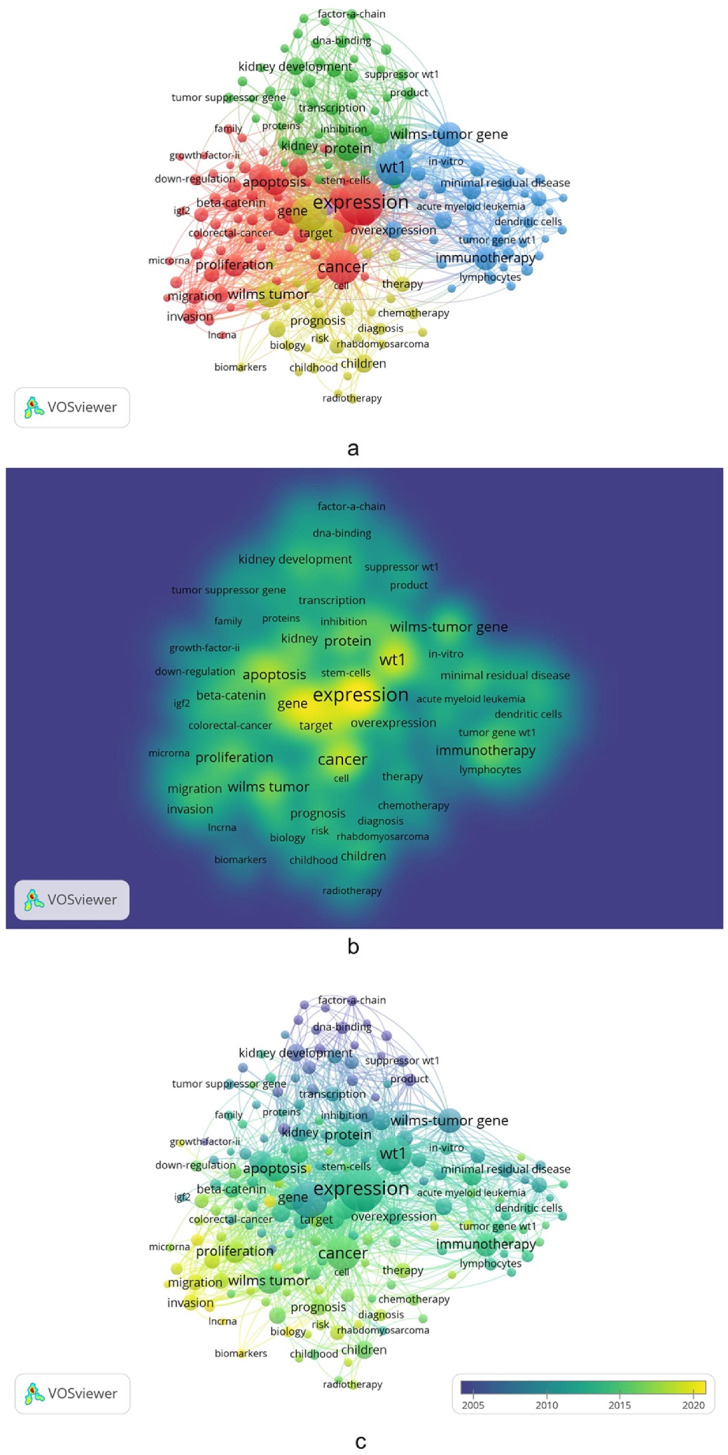
**a** Co-occurrence analysis of keywords relevant to WT targeted therapy. **b** Mapping on density visualization of co-occurrence analysis of keywords. Different colors stand for different frequencies of co-occurrence link strength. Blue indicates low frequency, green indicates medium frequency, and yellow indicates high frequency. Co-occurrence link strength in a yellow circle is more robust than those in other colored circles. **c** Visualization of the keyword appearing time according to overlay visualization. The keywords highlighted in yellow appeared later than those highlighted in green

A co-occurrence analysis of keywords can summarize topics of publications and identify connections between items by the frequency they arose together in papers [9, 19]. We used VOSviewer to reveal the prominent study key points and future directions in this field. The minimum occurrence frequency threshold of keywords was set as 15, and 178 nodes were acquired from a total of 6,674 keywords to generate co-occurrence network and density map, as displayed in Fig. 5a, b, respectively. The top three most frequently occurring keywords were “Expression” (469 occurrences), “Wilms tumor” (304 occurrences) and “WT1” (282 occurrences). While the terms “lncRNA”, “Biomarkers” and “microRNA” were the latest research hotspots according to overlay visualization map from VOSviewer, which may indicate future research directions (Fig. 5c).

## Discussion

In response to the burgeoning growth in multidisciplinary scholarship, it has become essential for researchers to possess a comprehensive understanding of the prevailing advancements within their respective areas of study. Different from conventional meta-analysis and systematic review approaches, bibliometric analysis offers a more objective and accessible method for validating and analyzing existing publications [20]. As far as we know, our research is the first bibliometric study to evaluate the present research activity and future trends of WT targeted therapy particularly. A total of 1,604 publications from the WoSCC database were analyzed to identify the research activity and future trends. Based on our analysis, the annual publications from 1987 to 2000 were relatively rare, indicating the research on WT targeted therapy was not enough. Publication volume and citation counts in WT targeted therapy research have surged since 2018, reflecting heightened scholarly focus on the biological and clinical heterogeneity of this domain. The total citations reached 64,800, which was higher than that for robotic arthroplasty area (*n* = 27,461) [21]. The discrepancy may be attributable to the size of the professional community and its respective interests [22].

With respect to the distribution of research, the USA dominated the research regarding the publication number, h-index and global collaboration network as expected, which was consistent with other bibliometric studies [23–25]. It was noteworthy that China, as the only developing country within the top ten nations in terms of publications, held the second position with 388 publications. This observation suggested a considerable investment in WT targeted therapy research, as well as an effective utilization of financial resources from China. However, despite China’s second place ranking in terms of overall publication number, the country exhibited deficiencies in the h-index, which indicated a potential for enhancement in the research quality and influence of Chinese scholars. Among the top ten institutions in terms of publication number, Harvard University contributed the most, while for the collaboration network of institutions, Osaka University was the dominant institution in this field. But the collaboration between these two top institutions were inadequate. Moreover, an obvious national boundary in the collaboration network of institutions and authors was found in our study, implying the degree of communication among institutions and authors from different countries was relatively insufficient. Domestically, academic collaborations are predominantly coordinated by key institutions, fostering multiple cooperative clusters. This core-periphery structure likely underpins the strong connectivity observed among national institutions and researchers [9]. However, at the global level, such collaboration requires further development. A worldwide analysis of cancer research funding revealed a huge imbalance regarding tumor study investment between high-income and low-income countries [26]. To this end, the promotion of continuous development of targeted therapy for WT is vital, necessitating encouragement of international collaboration, thus enabling involvement in interactive research by institutions and authors globally.

For the 1604 publications included in our analysis, *Oncogene* and *Blood* were the leaking sources of paper volume and co-citation, respectively, but they did not own the highest IF among all contributed journals. The discrepancy between the quality and quantity of research published in these journals and other high-level journals could be indicative of a research gap. An analysis of the geographical distribution in the top ten productive and co-cited journals revealed the USA had a dominant position in WT targeted therapy research. This phenomenon may be attributed to the existence of conducive environments for fundamental scientific research and clinical experimentation in the USA, characterized by the utilization of advanced technological resources, the presence of skilled researchers, and the allocation of sufficient financial resources. Additionally, previous studies have indicated that authors originating from the USA demonstrated a preference for the publication and citation of domestic articles [27, 28].

Information analysis of the impactful publications can provide valuable insights into the WT targeted research. In the present study, the top ten highly co-cited references were all published in high quality journals which were classified as Q1 by the 2023 journal citation report. The WT1 gene was the primary topic and the article with the highest co-citations was published in 1990 by Call KM et al. in *Cell*, with a total of 249 citations, which identified the gene of WT was located on human chromosome 11 by isolating a series of genomic and cDNA clones [18]. This study reflected the gene level research of WT targeted therapy significantly, which proved the critical function of WT1 in gonadal and renal development, along with tumorigenesis. The findings indicated the considerable clinical significance of WT1 in the context of targeted and genetic therapy.

Keyword co-occurrence analysis has been instrumental in elucidating the distribution of research hotspots within a given field. In keyword co-occurrence analysis, the frequent research hotspots in the field of WT targeted therapy were “Expression”, “Wilms tumor” and “WT1”. Several previous studies have pointed out the insulin-like growth factor 2 (IGF2) signaling pathway has been demonstrated to be closely associated with the development of WT. The combination of increased IGF2 gene expression and WT1 gene ablation has been shown to result in WT [29, 30]. At present, the IGF2 receptor is regarded as the most viable therapeutic target because of two key factors: over expression and its function in both the initiation and progression of WT [31]. When focusing on the future research trends, we found the terms “lncRNA”, “Biomarkers” and “microRNA” were the latest key points according to overlay visualization map from VOSviewer. Epigenetic factors have been an optional approach in cancer targeted therapy. lncRNAs, as epigenetic factors, play a crucial role in cancer initiation and development. MicroRNAs are known to be the most prominent targets of lncRNAs and the lncRNA-miRNA axis regulates cell death mechanisms, which performs an important part in therapy resistance of tumors [32, 33]. In addition, microRNAs have been shown to modulate the proliferation, migration and apoptosis of WT cells by affecting the protein expression via cell signaling pathways, which contributes significantly to the onset and progression of WT. Consequently, microRNAs have emerged as promising target sites for targeted drug development, offering potential therapeutic interventions for the treatment of WT [34–36].

This study remains several limitations. First, our literatures were retrieved only from the WoSCC database. While the WoSCC is globally prioritized for bibliometric studies due to its multidisciplinary coverage, citation analytics robustness and data integrity, its exclusion of non-indexed literature from specialized databases (e.g., PubMed, Scopus or Embase) may represent a potential limitation [14, 37]. Second, only papers published in English were included, which may underestimate the impact of non-English publications. Third, we exclusively included articles and reviews, while excluding other types of publications such as corrections or notes, although they may have potential value. Despite these, the present study still offered relatively objective information and insights, with the purpose of facilitating the WT targeted therapy research.

## Conclusions

The present study summarized the current research hotspots of targeted therapy research in WT and explored potential research trends comprehensively through bibliometric analysis. From the recent pattern, there will be a constant rise in the number of publications in the future, which underscores its growing importance in the scientific community. Nonetheless, a limited number of developed regions have dominated scientific outputs, and global collaboration remains inadequate. Hence, international collaborations and translational research should be enhanced to facilitate further advancements in this field.

## Data Availability

The data that supports the findings of this study is available from the corresponding author based on reasonable request.

## Abbreviations

IF: Impact factor
IGF2: Insulin-like growth factor 2
WT: Wilms tumor
WoSCC: Web of Science Core Collection
